# Differential effects of non-selective and cardio-selective beta-blocker therapy on ECG parameters in long QT syndrome type 1

**DOI:** 10.1016/j.ijcha.2026.101901

**Published:** 2026-03-11

**Authors:** Marina Rieder, Vanessa Christe, Saranda Nimani, Peter M. Deissler, Rachel M.A. ter Bekke, Katja E. Odening

**Affiliations:** a Translational Cardiology, Department of Cardiology and Institute of Physiology, Inselspital, Bern University Hospital and University of Bern, Bern, Switzerland; b Department of Cardiology, Maastricht University Medical Center+, Maastricht, Netherlands (the); c Cardiovascular Research Institute Maastricht (CARIM), Maastricht University, Maastricht, Netherlands (the); d Member of the European Reference Network for Rare, Low Prevalence, and Complex Diseases of the Heart - ERN GUARD-Heart, Netherlands (the)

## Abstract

• Non-selective β-blockers markedly reduce Tpeak/end in LQT1 patients.
• Cardio-selective β-blockers show no consistent effect on Tpeak/end.
• The nsBB effect persists after exercise, independent of heart rate.
• Tpeak/end shortening indicates decreased global repolarization heterogeneity and reduced arrhythmic risk.
• Tpeak/end may serve as a novel ECG marker for β-blocker efficacy in LQT1.

## 1 Introduction

Congenital long QT syndrome (LQTS) is caused by pathogenic variants in genes encoding cardiac ion channels involved in cardiac repolarization. This manifests as a prolonged heart rate-corrected QT interval (QTc) on the surface electrocardiogram (ECG) [1]. Affected patients face an increased risk of malignant ventricular arrhythmias, which can lead to syncope and/or sudden cardiac death [2]. The majority of patients harbor variants in in one of the three “major” LQTS genes *KCNQ1* (LQT1), *KCNH2* (LQT2), or *SCN5A* (LQT3), with *KCNQ1* accounting for approximately 40–55% of cases [3], [4]. *KCNQ1* encodes the α-subunit of the potassium channel Kv7.1, which underlies the slow delayed-rectifier repolarizing potassium current I_Ks_. I_Ks_ is enhanced upon adrenergic activation through phosphorylation, thereby shortening cardiac repolarization at higher heart rates [5]. Consequently, in case of genetically-impaired I_Ks_ like in LQT1, sympathetic surges may result in paradoxically prolonged cardiac repolarization / QTc and altered repolarization heterogeneity, predisposing these patients to arrhythmias during physical exertion or emotional stress [6], [7], [8].

Beta-blockers (BB), which aim to mitigate these pro-arrhythmic triggers, represent the cornerstone of LQTS therapy and significantly reduce mortality [9]. Non-selective beta-blockers (nsBB) are superior to cardio-selective beta-blockers (sBB) in preventing arrhythmic events [10]. Current guidelines recommend considering pharmacological treatment even in asymptomatic mutation carriers [11], but side effects and non-compliance remain significant challenges, and cardiac events might still occur despite treatment [9], [12]. Consequently, precise monitoring of therapeutic efficacy to identify patients at risk for arrhythmias despite treatment is critically important. Yet, commonly used parameters such as QTc at rest often fail to accurately predict individual arrhythmic risk [9], [11].

It is well established that not only the duration of repolarization but also its spatial and temporal heterogeneity plays a crucial role in arrhythmogenesis by causing functional conduction blocks and facilitating reentrant circuits [13], [14]. Notably, sympathetic activation can alter transmural heterogeneity of repolarization, acting not only as a trigger for arrhythmias but also modifying the arrhythmogenic substrate [15]. We previously described that ECG parameters, such as Tpeak/end or delta Tpeak/end (V5-V2), which are considered as surrogate markers for global dispersion of repolarization [16], may be superior to the classical QTc for risk stratification [17]. Therefore, we aimed to investigate the BB effect on these electrical parameters.

## 2 Materials and methods

The study population consisted of patients with genetically confirmed LQT1 presenting at the Genetic Arrhythmia Clinic at the University Hospital of Bern (Inselspital), Switzerland or the Maastricht University Medical Center+, the Netherlands.

These patients were referred for genetic counseling, testing or routine clinical consultations. The decision to perform examinations (12-lead ECGs / exercise stress tests) or to start specific medication was solely at the discretion of the attending physician and not conducted for study purposes.

The analysis was approved by the Cantonal Ethics Committee of Bern (ID 2016-01602 and ID 2020-00316) and the Ethics Review Committee MUMC+/UM (METC 19-066 and NCT04976348). All participants had provided written informed consent.

### 2.1 Study population

Only patients with a pathogenic or likely pathogenic variant in *KCNQ1,* and thus genetically confirmed LQT1, were included in the analysis. Variant interpretation followed the principles and recommendations of the statement of the American College of Medical Genetics and Genomics/Association of Molecular Pathology [18]. Only patients in whom 12-lead ECGs at rest and exercise stress tests were performed both with and without BB therapy were included. Standard 12-lead ECGs were recorded at 25 mm/s using standard lead positions, with a 10-second recording duration at rest. Exercise testing was performed according to the standard or modified Bruce protocol, depending on the patient‘s physical capacity [19]. Patients with a history of presumably cardiac syncope, presyncope, and/or documented torsade de Pointes (TdP) tachycardia were classified as symptomatic. None of the patients had a history of aborted cardiac arrest.

### 2.2 Assessment of electrical parameters

QT and RR intervals, along with the corresponding heart rates, were assessed in lead II, as this is the lead of choice to determine QTc in LQTS [20]. The QT interval was measured from the onset of the QRS complex to the end of the T wave, which was determined using the tangent method [21]. QT intervals were corrected for heart rate using Bazett’s formula [22], supported by evidence from genetically confirmed LQTS patients, as it most effectively minimizes QT–heart rate dependency and provides the most consistent diagnostic and prognostic classification compared with alternative correction methods [23]. No patients in our study had a prolonged QRS complex or bundle branch block.

Additionally, the interval between the peak and end of the T wave (Tpeak/end), also determined by the tangent method, was assessed in leads V2 and V5. Lead V5 was chosen according to previous LQTS studies because it overlies the left ventricular lateral wall, where the transmural dispersion of repolarization seems to be greatest in LQTS, while also offering a stable and well-defined T-wave morphology for reliable measurement and has been consistently used in previous studies [1], [24], [25]. Lead V2 was included to capture repolarization in the right ventricular region, which can provide complementary information on regional repolarization differences [24]. Similar to the QT interval, the Tpeak/end interval was corrected for heart rate using Bazett’s formula (Tpeak/end corrected = Tpeak/end / √RR) to account for the dependency of repolarization duration on heart rate, ensuring more accurate and comparable assessments across different heart rates. Furthermore, the novel parameter delta Tpeak/end (Tpeak/end in V5 minus Tpeak/end in V2) was calculated [17].

All electrocardiographic analyses were performed at rest (recorded in a supine position) and during the exercise stress test (before start of exercise in an upright position and at minute 2 of the post-exercise recovery phase). All parameters were measured over three consecutive beats, and averaged. The measurements were performed manually by a board certified cardiologist (M.R.). To assess the interobserver variability, QTc and heart rate corrected Tpeak/end in lead V5 from a subset of patients were independently determined by another board certified cardiologist (V.C.). The degree of agreement between the two observers was subsequently analyzed to ensure measurement reliability.

### 2.3 Statistical analysis

Statistical analyses were performed using GraphPad Prism 10 (GraphPad Software, San Diego, CA, USA) and IBM SPSS Statistics (IBM Corp., Armonk, NY, USA).

Data are presented as mean ± standard deviation. The ROUT method was applied with consecutive exclusion of significant outliers. Data normality was assessed using the Shapiro–Wilk test. To compare electrical parameters before and after β-blocker therapy, mixed-effects analysis for repeated measures with Dunnett’s multiple comparison test was performed. Pearson’s correlation coefficients were calculated to evaluate associations between Tpeak/end and heart rate or QTc. Inter-rater reliability for QTc and heart rate corrected Tpeak/end measurements was assessed using the intraclass correlation coefficient (ICC) based on a two-way mixed-effects model with absolute agreement and single measurements. Statistical significance of the ICC was determined using an F-test. Significance threshold was set at p < 0.05.

## 3 Results

### 3.1 Patient population

A total of 16 patients with genetically confirmed LQT1 (mean age of 34 ± 18 years) were included in the analysis (9/16 female, 7/16 male, 6/16 symptomatic) with the following pathogenic or likely pathogenic variants in *KCNQ1* (NM_000218.3): c.1097G > A (p.Arg366Gln; 1 patient), c.745A > G (p.Ile249Val; 1 patient), c.691C > T (p.Arg231Cys; 5 patients), c.1685 + 2T > G (p.(?); 1 patient), c.(449_527)_(860_959)del (p.(?); 1 patient), c.1189C > T (p.Arg397Trp; 1 patient), c.477 + 5 G > A (p.(?); 1 patient), c.1664G > A (p.Arg555Gln; 1 patient), c.1760C > T p.(Thr587Met; 2 patients), c.1772G > A (p.Arg591His; 1 patient), c.724G > A (p.D242N; 1 patient). Further details are listed in Suppl. Table 1.

### 3.2 12-lead ECG at rest

ECG data prior to initiation of BB therapy was available for n = 15 patients. Fourteen of these patients had ECGs available while on maximum tolerated dose of nsBB treatment (eight patients on propranolol, six on nadolol) and ten had ECGs while on sBB treatment (one on nebivolol, six on metoprolol, two on bisoprolol, one on atenolol). In total, seven patients had ECGs available beta-blocker naïve and for both sBB and nsBB. Further details see Suppl. Table 1.

**Fig. 1:**
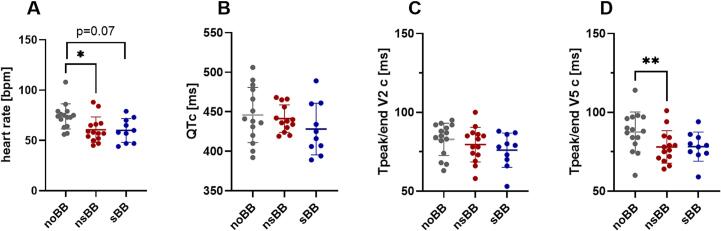
**12-lead ECG parameters at rest (supine position).** ECG measurements are shown for three conditions: prior to beta-blocker therapy (noBB, grey; n = 15), during treatment with a non-cardioselective beta-blocker (nsBB, red; n = 14), and during treatment with a cardioselective beta-blocker (sBB, blue; n = 10). Complete ECG data across all three conditions were available for n = 7 participants. * p < 0.05; **p < 0.01 (mixed-effect analysis for repeated measures with Dunnett’s multiple comparison test). Data are shown as individual values with mean ± SD. **(A)** Heart rate [bpm] was significantly lower after nsBB intake compared to the heart rate prior to beta-blocker therapy (p = 0.01) and tended to be lower after sBB intake. **(B)** The heart rate-corrected QT interval (QTc) showed no significant differences between groups. **(C)** The heart rate-corrected Tpeak/end interval in lead V2 (Tpeak/end V2c) did not differ significantly between groups. **(D)** The heart rate-corrected Tpeak/end interval in lead V5 (Tpeak/end V5c) was significantly reduced after nsBB intake compared to values prior to therapy (p = 0.001). (For interpretation of the references to colour in this figure legend, the reader is referred to the web version of this article.)

In the 12-lead ECGs obtained in the supine position, the heart rate was significantly lower after initiation of nsBB (61 ± 13 bpm with nsBB (p = 0.01) vs. 74 ± 13 bpm without BB) and tended to be lower after initiation of sBB (60 ± 12 bpm, p = 0.07, Fig. 1A).

Prior to initiation of BB therapy, QTc was 446 ± 35 ms. After initiation of BB therapy, QTc was lower, but not significantly different (441 ± 17 ms with nsBB (p = 0.86) and 428 ± 33 ms with sBB (p = 0.14)) (Fig. 1B; RR and QT-interval durations are depicted in Suppl. Fig. 1A and B).

Similar effects were observed for Tpeak/end (heart rate-corrected) in lead V2, with no significant differences before and after BB initiation (80 ± 11 ms with nsBB vs. 83 ± 10 ms without BB (p = 0.58) and 76 ± 11 ms with sBB (p = 0.25), Fig. 1C). However, in lead V5, Tpeak/end (heart rate-corrected) was significantly reduced after initiation of nsBB (78 ± 10 ms with nsBB vs 88 ± 13 ms without BB (p = 0.001)), but not after sBB therapy (78 ± 9 ms, p = 0.12, Fig. 1D).

There was no statistical difference in delta Tpeak/end (heart rate-corrected, V5-V2) after initiation of BB (5 ± 13 ms without BB vs. −1 ± 14 ms with nsBB (p = 0.35) and 2 ± 10 ms with sBB (p = 0.83)).

To determine whether the reduction in Tpeak/end in lead V5 was attributable to the decreased heart rate following initiation of nsBB, we calculated the Pearson correlation coefficient between heart rate and heart rate–corrected Tpeak/end in V5 in the nsBB cohort. The correlation coefficient (r) was −0.16 (p = 0.6), suggesting no significant association. These results indicate that the observed changes in Tpeak/end (heart rate–corrected) in V5 were independent of the reduction in heart rate in the nsBB cohort (Suppl. Fig. 1C).

**Fig. 2:**
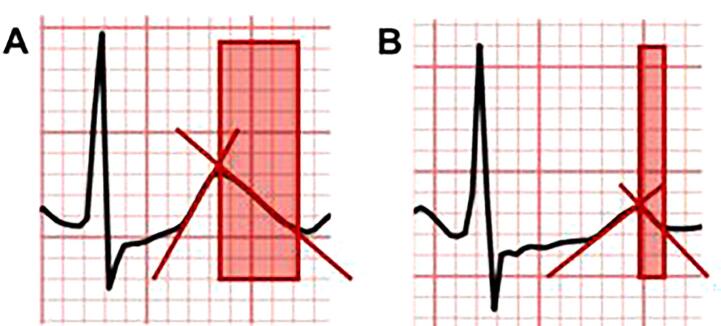
**Example of Tpeak/end reduction with non-cardioselective beta-blocker therapy.** Tpeak/end interval in ECG lead V5 (highlighted by red box) recorded two minutes after exercise in a patient with LQT1 (KCNQ1 c.1685 + 2T > G). The ECG was recorded at a paper speed of 25 mm/sec and a voltage scale of 10 mm/mV. **(A)** Tpeak/end in lead V5 prior to beta-blocker therapy. **(B)** Tpeak/end in lead V5 while on non-cardioselective beta-blocker therapy with nadolol (1 mg/kg body weight). (For interpretation of the references to colour in this figure legend, the reader is referred to the web version of this article.)

As an exemplary illustration of beta-blocker effects on Tpeak/end, Fig. 2 shows Tpeak/end interval shortening in lead V5 in one of our patients after administration of the non-cardioselective beta-blocker nadolol.

Next, we aimed to evaluate whether these findings applied to both the symptomatic and the asymptomatic cohort.

**Fig. 3:**
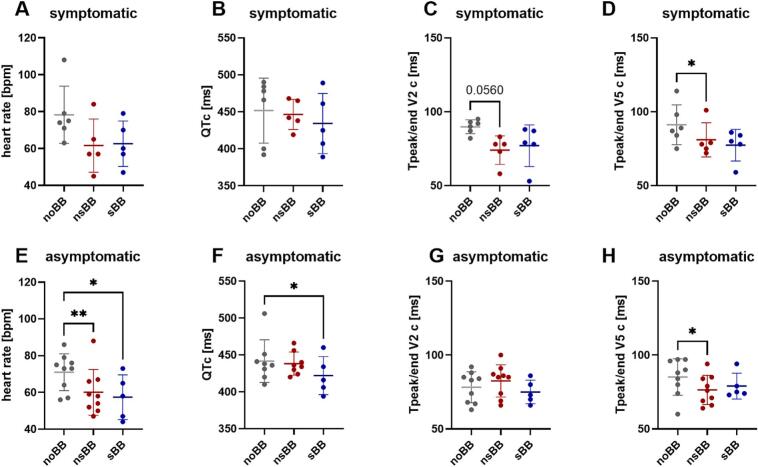
**12-lead ECG parameters at rest (supine position), symptomatic / asymptomatic cohort** A-D: symptomatic patients: ECG measurements are shown for three conditions: prior to beta-blocker therapy (noBB, grey; n = 6), during treatment with a non-cardioselective beta-blocker (nsBB, red; n = 5), and during treatment with a cardioselective beta-blocker (sBB, blue; n = 5). Complete ECG data across all three conditions were available for n = 4 participants. **(A)** heart rate was not significantly altered by BB treatment **(B)** The heart rate-corrected QT interval (QTc) showed no significant differences between groups. **(C)** The heart rate-corrected Tpeak/end interval in lead V2 (Tpeak/end V2c) tended to be shorter after nsBB intake **(D)** The heart rate-corrected Tpeak/end interval in lead V5 (Tpeak/end V5c) was significantly reduced after nsBB intake compared to values prior to therapy (p = 0.03). **E-H: asymptomatic patients:** ECG measurements are shown for three conditions: prior to beta-blocker therapy (noBB, grey; n = 9), during treatment with a non-cardioselective beta-blocker (nsBB, red; n = 8), and during treatment with a cardioselective beta-blocker (sBB, blue; n = 5). Complete ECG data across all three conditions were available for n = 3 participants. **(A)** heart rate was significantly lowered by nsBB (p = 0.005) and sBB treatment (p = 0.03) **(B)** The heart rate-corrected QT interval (QTc) was lower after sBB intake (p = 0.02) **(C)** The heart rate-corrected Tpeak/end interval in lead V2 (Tpeak/end V2c) was not altered by BB therapy **(D)** The heart rate-corrected Tpeak/end interval in lead V5 (Tpeak/end V5c) was lower after nsBB intake (p = 0.05) *p < 0.05, ** p < 0.01 (mixed-effects analysis for repeated measures with Dunnett’s multiple comparison test). Data are shown as individual values with mean ± SD. (For interpretation of the references to colour in this figure legend, the reader is referred to the web version of this article.)

In the symptomatic cohort, neither heart rate (78 ± 15 ms without BB vs. 62 ± 14 ms with nsBB (p = 0.3) and 63 ± 12 ms with sBB (p = 0.38), Fig. 3A) nor QTc (452 ± 44 ms without BB vs. 446 ± 20 ms with nsBB (p = 0.93) and 434 ± 41 ms with sBB (p = 0.57), Fig. 3B) were significantly affected by BB therapy. Tpeak/end (heart-rate corrected) tended to be shorter in lead V2 after initiation of nsBB, but not sBB (90 ± 5 ms without BB vs. 74 ± 10 ms with nsBB (p = 0.06) and 77 ± 14 ms with sBB (p = 0.24), Fig. 3C) and was significantly shorter in lead V5 with nsBB (91 ± 13 ms without BB vs. 81 ± 12 ms with nsBB (p = 0.03) and 77 ± 11 ms with sBB (p = 0.24), Fig. 3D).

In the asymptomatic cohort, QTc was reduced after initiation of sBB, but not nsBB therapy (442 ± 29 ms without BB vs. 438 ± 16 ms with nsBB (p = 0.95) and 422 ± 26 ms with sBB (p = 0.02), Fig. 3F). Heart rate was significantly lower after nsBB (71 ± 10 ms prior to BB therapy vs. 60 ± 12 ms with nsBB (p = 0.005) and 57 ± 12 ms with sBB (p = 0.03), Fig. 3E).

Heart-rate corrected Tpeak/end was not affected by BB therapy in V2 (78 ± 10 ms without BB vs. 83 ± 11 ms with nsBB (p = 0.36) and 75 ± 8 ms with sBB (p = 0.76), Fig. 3G) but reduced by nsBB in V5 (85 ± 12 ms without BB vs. 76 ± 10 ms with nsBB (p = 0.05) and 79 ± 9 ms with sBB (p = 0.54, Fig. 3H).

### 3.3 Exercise-stress test

To evaluate whether the reduction in transmural repolarization heterogeneity with nsBB therapy persisted during exercise, we evaluated ECG parameters prior to exercise (standing position) and at minute two of the recovery period.

**Fig. 4:**
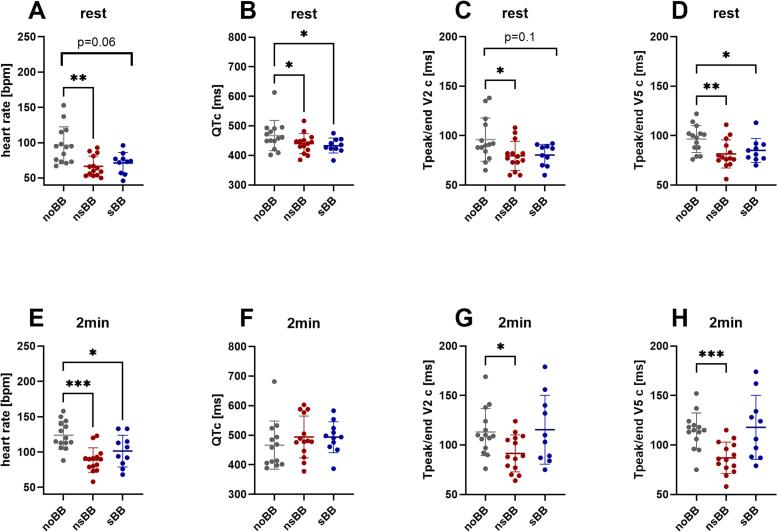
**ECG parameters during an exercise stress test at rest (standing position) and at minute two of the recovery period. A-D**: ECG measurements prior to exercise (standing position) are shown for three conditions: without beta-blocker treatment (noBB, grey; n = 14), during treatment with a non-selective beta-blocker (nsBB, red; n = 14), and during treatment with a selective beta-blocker (sBB, blue; n = 10). Complete ECG data across all three conditions were available for n = 6 participants. **(A)** heart rate was significantly altered by nsBB treatment (p = 0.003) and tended to be lower after sBB treatment (p = 0.06). **(B)** The heart rate-corrected QT interval (QTc) was lower after nsBB (0.05) and sBB (0.03) intake **(C)** The heart rate-corrected Tpeak/end interval in lead V2 (Tpeak/end V2c) was lower after nsBB intake (p = 0.02) **(D)** The heart rate-corrected Tpeak/end interval in lead V5 (Tpeak/end V5c) was significantly reduced after nsBB (p = 0.006) and sBB (p = 0.02) intake **E-H**: ECG measurements at minute two of the recovery period for the same groups as above. **(E)** heart rate was significantly altered by nsBB (p < 0.001) and sBB (p = 0.03)treatment two minutes after exercise **(F)** The heart rate-corrected QT interval (QTc) two minutes after exercise showed no significant differences between groups. **(G)** The heart rate-corrected Tpeak/end interval in lead V2 (Tpeak/end V2c) two minutes after exercise was shorter after nsBB intake (p = 0.02). **(H)** The heart rate-corrected Tpeak/end interval in lead V5 (Tpeak/end V5c) two minutes after exercise was significantly reduced after nsBB intake (p < 0.001). *p < 0.05, ** p < 0.01 ***p < 0.001; mixed-effects analysis for repeated measures with Dunnett’s multiple comparison test.Data are shown as individual values with mean ± SD. (For interpretation of the references to colour in this figure legend, the reader is referred to the web version of this article.)

Heart rate was reduced after both nsBB and sBB therapy at rest and after two minutes of recovery. At rest, heart rate decreased from 96 ± 26 bpm without BB to 66 ± 15 bpm with nsBB (p = 0.003) and to 71 ± 15 bpm with sBB (p = 0.06); Fig. 4A. After two minutes of recovery, heart rate was 124 ± 20 bpm without BB, 89 ± 17 bpm with nsBB (p < 0.001), and 102 ± 23 bpm with sBB (p = 0.03); Fig. 4E.

QTc was lower after initiation of BB therapy, prior to exercise (467 ± 51 ms noBB vs. 440 ± 34 ms with nsBB (p = 0.05) and 433 ± 25 ms with sBB (p = 0.03); Fig. 4B) but not two minutes post-exercise (467 ± 81 ms noBB vs. 494 ± 71 ms with nsBB (p = 0.15) and 493 ± 52 ms with sBB (p = 0.21); Fig. 4F).

In lead V2, Tpeak/end was significantly lower after nsBB treatment at rest (96 ± 22 ms without BB vs. 80 ± 14 ms with nsBB, p = 0.02), while no significant change was observed with sBB (80 ± 11 ms, p = 0.10; Fig. 4C). At two minutes of the recovery period, Tpeak/end V2 (heart rate corrected) was again reduced with nsBB (113 ± 24 ms without BB vs. 92 ± 19 ms with nsBB, p = 0.02), whereas no effect was seen with sBB (116 ± 35 ms, p = 0.75; Fig. 4G).

In lead V5, nsBB therapy consistently reduced Tpeak/end. At rest, Tpeak/end decreased with both nsBB (97 ± 14 ms without BB vs. 82 ± 14 ms with nsBB, p = 0.01) and sBB (85 ± 12 ms, p = 0.02; Fig. 4D). However, after two minutes of recovery, the reduction persisted only with nsBB (114 ± 18 ms without BB vs. 87 ± 16 ms with nsBB, p < 0.001), whereas sBB had no effect (118 ± 32 ms, p = 0.87; Fig. 4H).

Overall, nsBB showed a consistent and robust effect across both leads and conditions, whereas the effects of sBB were less uniform.

Delta Tpeak/end (heart rate-corrected, V5-V2) remained unchanged at all time points (Suppl. Fig. 2A and 2B).

We calculated the correlation coefficients between heart rate and heart rate–corrected Tpeak/end at rest and two minutes after exercise in the nsBB cohort. At rest, the Pearson correlation coefficient was r = 0.3 (p = 0.3) for Tpeak/end V2 (heart rate–corrected) and r = 0.2 (p = 0.49) for Tpeak/end V5 (heart rate–corrected) (Suppl. Fig. 3A and 3B). Two minutes post exercise, the correlation coefficients were r = 0.12 (p = 0.68) for both Tpeak/end V2 and r = 0.26 (p = 0.46) for Tpeak/end V5 (heart rate–corrected) (Suppl. Fig. 3C and 3D). These results indicate that the heart rate–corrected Tpeak/end values were not significantly associated with heart rate at either time point. Therefore, the observed reduction in Tpeak/end after initiation of nsBB appears to be independent of the nsBB-associated reduction in heart rate.

### 3.4 Reproducibility

To assess the interobserver variability, QTc and heart rate corrected Tpeak/end in lead V5 from a subset of patients were independently determined by two cardiologists. The intraclass correlation coefficient (ICC) for QTc measurements between the two raters was 0.889 (95% confidence interval [CI]: 0.400 to 0.984), indicating excellent inter-rater reliability. This agreement was statistically significant (F(5,5) = 23.466, p = 0.002). Likewise, the ICC for heart rate-corrected Tpeak/end in lead V5 was 0.788 (95% CI: 0.056 to 0.968), demonstrating good reliability, with the corresponding F-test reaching statistical significance (F(5,5) = 7.416, p = 0.023). These findings indicate that the two raters produced comparable and consistent measurements for QTc and heart rate corrected Tpeak/end.

## 4 Discussion

In this study, we demonstrated that the global dispersion of repolarization, as assessed by the heart rate–corrected Tpeak/end interval on 12-lead ECG [16], was significantly reduced following initiation of non-selective beta-blocker (nsBB) therapy in patients with LQT1. Exercise-related shortening of Tpeak/end has also been described in healthy individuals, and in our cohort nsBB therapy induced a similar pattern, consistent with a partial normalization of repolarization [26]. This effect was consistent across different settings, further enhanced during exercise, and occurred independently of heart rate reduction. Selective beta-blocker (sBB) therapy showed a reduction in Tpeak/end in some isolated settings (e.g., in lead V5 or during exercise), but these effects were less consistent and not maintained during recovery. Taken together, our findings suggest that nsBB therapy exerts a more robust and clinically relevant modulation of repolarization heterogeneity compared to sBBs.

Beta-blocker therapy remains the cornerstone of management in LQTS, significantly decreasing the incidence of cardiac events [9], [27]. However, the risk of recurrent arrhythmias remains substantial, particularly among patients who were symptomatic prior to treatment [9]. The rates of breakthrough events reported in earlier studies are likely overestimated, as these studies did not differentiate between patients treated with non-selective beta-blockers (nsBB) and those receiving selective beta-blockers (sBB) [9], since the superiority of non-selective preparations in LQTS was only discovered later [10]. Nevertheless, despite adequate beta-blocker therapy, breakthrough events of ventricular arrhythmia may still occur [28].

Nowadays, it is generally accepted that non-selective beta-blockers are superior in preventing arrhythmic events in LQTS, as also reflected in the 2022 ESC guideline recommendations [11].

The superiority of non-selective compared to cardio-selective beta-blockers in LQTS is on the one hand due to the additional Na^+^ channel blocking effect of the non-selective preparations, which metoprolol, for example, as a selective beta-blocker, does not have [10], [29]. As late Na^+^ channel blockers can also convey small action potential duration (APD)-shortening effects in LQT1 and LQT2 [30], [31], this may contribute to the more pronounced anti-arrhythmic effects. In addition, non-selective beta-blockers also inhibit beta 2 and beta 3 receptors [32]. Activation of beta 2 receptors was associated with increased arrhythmogenesis in a guinea pig model of heart failure and beta 3 receptors are known to be involved in the phosphorylation and activation of KCNQ1/I_Ks_ current [33], [34]. Therefore, by blocking these additional receptor subtypes, nsBBs may further suppress pro-arrhythmic triggers.

Propranolol is the most widely used nsBB. Its main disadvantage is the short half-life of only 3–6 h, necessitating thrice-daily dosing. Furthermore, the available extended-release formulation is only available in a fixed dose, which may limit dose flexibility and make it unsuitable for some patients requiring tailored dosing regimens. An alternative to propranolol is the longer-acting nsBB nadolol, which only needs to be taken once daily due to its longer half-life [35]. Unfortunately, nadolol is not available in many countries, including Switzerland, where it can only be obtained as an imported drug after approval from the health insurance company [36]. Due to these difficulties in providing patients with nadolol and the high frequency of intake needed in propranolol, one patient in this cohort unfortunately had to be treated with metoprolol.

In patients with LQTS, the repolarization duration (QTc) is commonly used as a marker for arrhythmogenic risk in clinical practice [11]. However, QTc is not suitable for assessing the therapeutic effect of BB or the arrhythmogenic risk under BB therapy, as most studies report either minimal or non-significant changes in QTc during BB therapy [9], [37], [38], [39], while others show diverse effects depending on underlying heart rate [40]. In line with these findings, we observed a reduction in QTc under sBB therapy only at standing position prior to exercise.

Not only the overall duration of repolarization but also its spatial heterogeneity contributes to the so-called “arrhythmogenic substrate” and plays an important role in the development of reentry-based arrhythmias [14]. Studies investigating myocardial repolarization heterogeneity have shown that differences in action potential duration across myocardial cell types and regions—such as epicardial, midmyocardial, and endocardial layers—underlie the inscription of the T wave on the surface ECG, and that these gradients are reflected in regional markers such as the Tpeak/end interval on different ECG leads [41], [42], [43]. Accordingly, the Tpeak/end interval on the ECG has been proposed as a surrogate marker for the dispersion of ventricular repolarization. It is thought to reflect differences in action potential duration (APD) across the ventricular wall, with the peak of the T-wave corresponding to the end of epicardial repolarization and the end of the T-wave corresponding to the completion of repolarization of the latest repolarizing cells—often attributed to M cells located in the midmyocardial layer [44], [45]. However, this concept—particularly the existence and role of M cells in the intact heart—remains debated and has been challenged in experimental and computational studies [46]. Therefore, the Tpeak/end interval should be interpreted with caution and is best considered a surrogate marker for global dispersion of repolarization rather than a direct measurement of transmural heterogeneity [16]. Nonetheless, increased Tpeak/end duration has been associated with a higher risk of sudden cardiac death in both congenital and acquired forms of LQTS [25], [47], [48], [49], [50].

So far, diverging findings have been reported on the influence of BB therapy on Tpeak/end. It was first described that propranolol reduces Tpeak/end [51]. Another study described a reduction of Tpeak/end during exercise in a cohort of 10 LQT1 patients [52], while another study of 23 LQTS patients with faster heart rates reported no Tpeak/end changes [40]. Notably, both studies included patients on both nsBB and sBB therapy. However, in the study of Gemma et al., [52], most patients received nsBB, which likely explains the observed reduction in Tpeak/end under BB therapy, similarly to our findings comparing nsBB vs sBB. In contrast, Bennett et al. evaluated patients on the sBB bisoprolol, which may explain the lack of change in Tpeak/end under BB therapy [40], similarly as in our patients treated with sBB. In our previous study on ECG parameters in LQTS patients, we found an association between arrhythmogenic risk and Tpeak/end in LQT2 patients at rest, but not in LQT1 [17]. However, that cohort included patients with and without BB (selective and non-selective), so the results are not directly comparable to those of the current study.

To date, there is no consensus on the optimal method for monitoring the therapeutic efficacy of beta-blocker (BB) therapy in patients with LQTS. While target doses for non-selective BBs such as propranolol and nadolol are recommended, achieving these dosages in clinical practice can be challenging. Contraindications—including asthma—and side effects such as fatigue and exercise intolerance often limit up-titration. Consequently, premature discontinuation of therapy is not uncommon and may increase the risk of life-threatening breakthrough arrhythmic events [27]. Therefore, the identification of novel ECG-derived parameters that reliably reflect therapeutic response is of critical importance. In light of our observation that Tpeak/end duration significantly shortens with non-selective beta-blocker (nsBB) therapy, we propose that Tpeak/end may serve as a promising candidate marker for monitoring treatment efficacy. Nonetheless, this hypothesis requires validation in larger, prospective patient cohorts.

## 5 Conclusion and outlook

We observed a reduction in Tpeak/end, a surrogate marker for global dispersion of repolarization, in patients with LQT1 treated with nsBB therapy. This effect was already present at rest and became more pronounced during and after exercise, consistent with the pathophysiology of LQT1, in which impaired repolarization is particularly unmasked under conditions of adrenergic stimulation [4]. In contrast, sBB therapy showed only occasional reductions in Tpeak/end, without consistent or sustained effects across all leads and conditions.

Given that nsBBs — which are associated with Tpeak/end shortening — are clinically more effective in preventing arrhythmic events than sBBs, which do not consistently reduce Tpeak/end, we propose that Tpeak/end may represent a promising surrogate marker for monitoring the therapeutic efficacy of nsBB treatment in LQTS. However, this hypothesis warrants confirmation in larger, prospective, and ideally multinational cohorts.

Taken together, our findings suggest that nsBB therapy confers distinct anti-arrhythmic benefits in LQT1 not only through a reduction of pro-arrhythmic triggers but also through the modulation of spatial repolarization gradients. This mechanistic insight may help explain the superior clinical outcomes associated with nsBBs compared to sBBs in this patient population.

## 6 Limitations

Our study cohort was relatively small, primarily because only a limited number of LQT1 patients underwent exercise stress testing prior to the initiation of beta-blocker (BB) therapy. In most cases, referring centers based their initial diagnostic work-up on resting surface ECGs, with BB therapy subsequently initiated without further functional testing. Furthermore, discontinuation of established therapy for research purposes is not ethically justifiable, further limiting the size of a treatment-naïve cohort.

Nevertheless, despite its limited sample size, our study provides valuable insight that may help reconcile previously conflicting findings. While one study reported BB-induced Tpeak/end shortening in LQTS patients [52], another found no such effect [40]. By directly comparing the effects of non-selective and selective BB therapy on ECG parameters, we were able to demonstrate that only nsBBs significantly shorten Tpeak/end. Notably, this effect was most pronounced in patients who were symptomatic prior to treatment.

These findings underscore the potential of Tpeak/end as a non-invasive marker for monitoring the therapeutic efficacy of BB therapy — particularly nsBBs — in LQTS. Further investigation in larger patient cohorts with longitudinal follow-up is warranted to validate the clinical utility of Tpeak/end in this context, while addressing the limitations of our relatively small sample size and the associated risk of type II error.

**Funding**

This study was funded by a grant from the “Gottfried and Julia Bangerter-Rhyner-Stiftung” Switzerland, a grant from the University Hospital of Bern, Inselspital (“Nachwuchsförderungs-Grant”) to MR, and a grant from the Bern Center of Precision Medicine to KEO. PD was supported by the German Academic Scholarship Foundation and the German Research Foundation (Walter Benjamin Programme, 529532291). RtB received grants from The Netherlands Organization for Scientific Research (Veni grant, 0915016181013), and the Health Foundation Limburg, Maastricht.

## CRediT authorship contribution statement

**Marina Rieder:** Conceptualization, Data curation, Formal analysis, Methodology, Project administration, Writing – original draft. **Vanessa Christe:** Data curation, Investigation, Writing – review & editing. **Saranda Nimani:** Writing – review & editing. **Peter M. Deissler:** Data curation, Investigation, Writing – review & editing. **Rachel M.A. ter Bekke:** Data curation, Resources, Writing – review & editing. **Katja E. Odening:** Conceptualization, Investigation, Project administration, Resources, Supervision, Writing – review & editing.

## Declaration of competing interest

The authors declare that they have no known competing financial interests or personal relationships that could have appeared to influence the work reported in this paper.
