# Supplementary figures and images for: Differential effects of non-selective and cardio-selective beta-blocker therapy on ECG parameters in long QT syndrome type 1
Source: Int J Cardiol Heart Vasc. 2026 Mar 11;64:101901. doi: 10.1016/j.ijcha.2026.101901 (PMC12996241; doi:10.1016/j.ijcha.2026.101901)

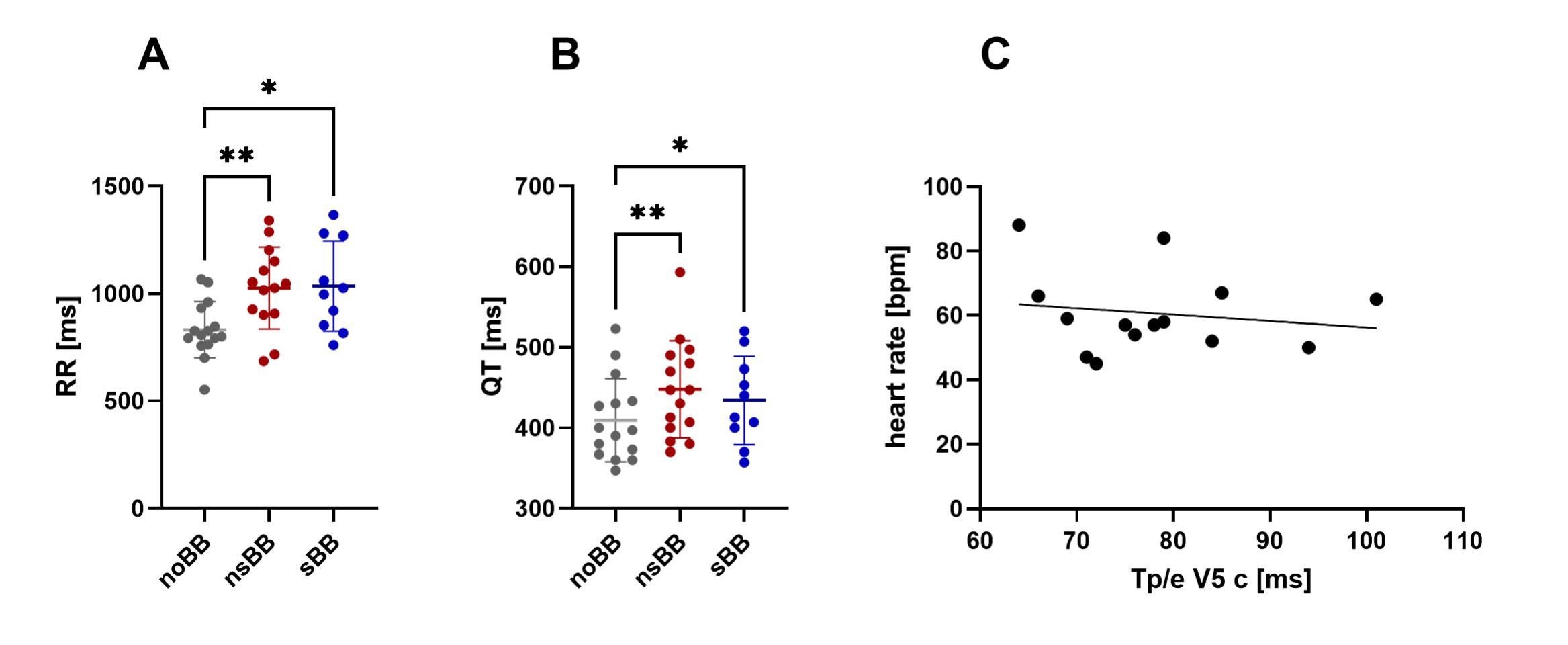

Supplement: Supplementary Figure 1 [file mmc1.jpg]

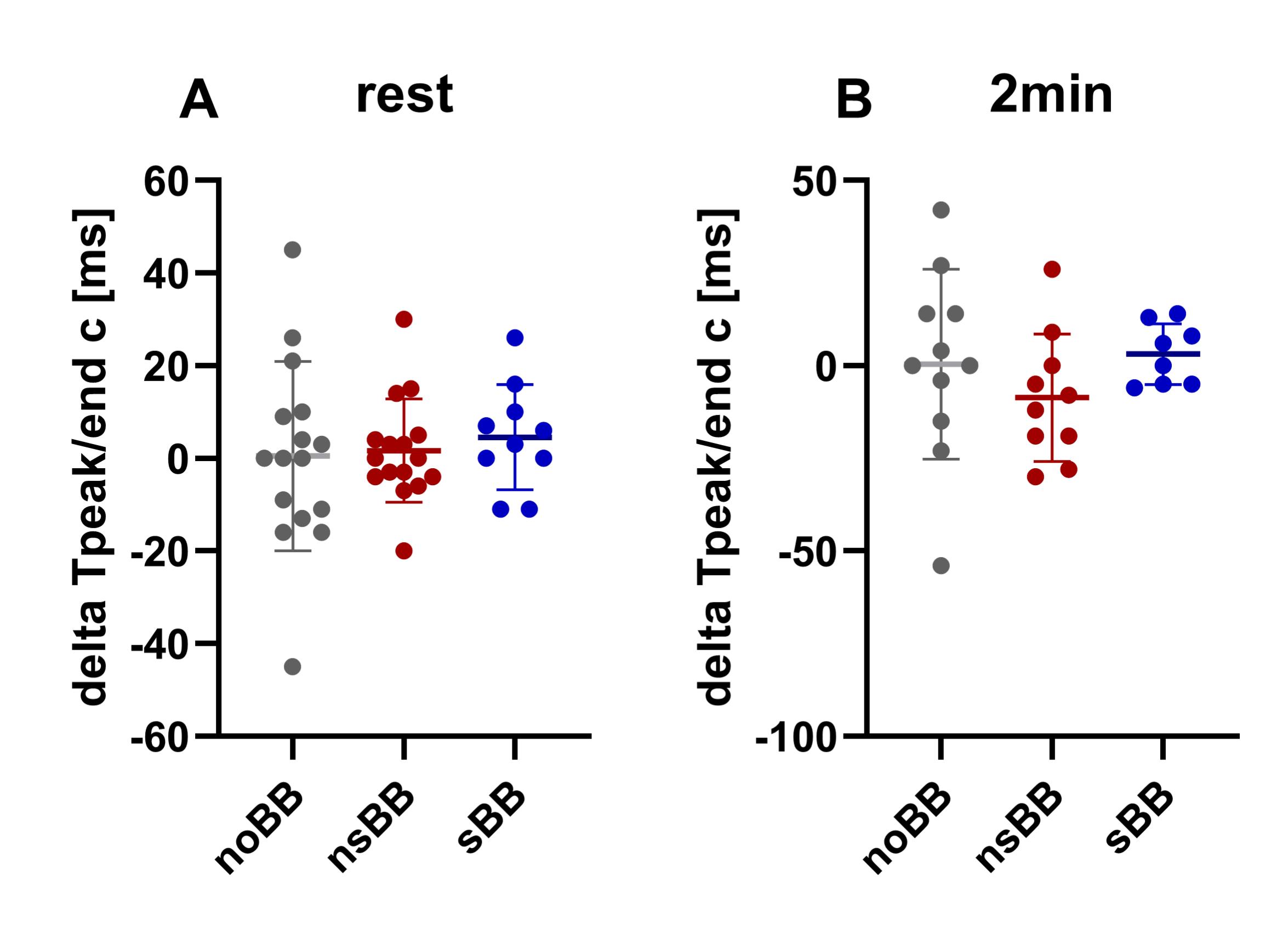

Supplement: Supplementary Figure 2 [file mmc2.jpg]

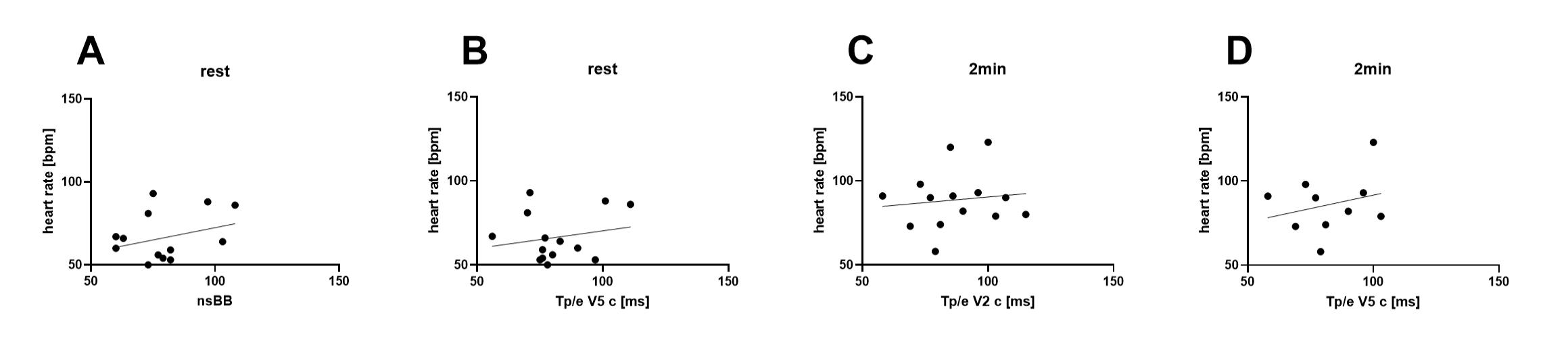

Supplement: Supplementary Figure 3 [file mmc3.jpg]
