# Supplementary material for: Differential effects of non-selective and cardio-selective beta-blocker therapy on ECG parameters in long QT syndrome type 1
Source: Int J Cardiol Heart Vasc. 2026 Mar 11;64:101901. doi: 10.1016/j.ijcha.2026.101901 (PMC12996241; doi:10.1016/j.ijcha.2026.101901)
Supplement: Supplementary Data 4 [file mmc4.docx]

**Supplement**

**Table 1**.

| **Patient** | **Sex** | **KCNQ1 variant** | **proband / family** | **Symptoms** | **non-selective BB** | **selective BB** |
| --- | --- | --- | --- | --- | --- | --- |
| 1 | female | c.1097G>A | family | symptomatic VT | nadolol (0.5mg/kgBW) | - |
| 2 | female | c.745A>G | proband | recurrent syncope | propranolol (2mg/kgBW) | nebivolol 2.5mg/d |
| 3 | male | c.691C>T | family | early onset atrial fibrillation | nadolol (1mg/kgBW) | metoprolol 50mg/d |
| 4 | female | c.691C>T | family | presyncope | nadolol (1mg/kgBW) | metoprolol 25mg/d |
| 5 | female | c.691C>T | family | asymptomatic | propranolol (1.4mg/kgBW) |  |
| 6 | male | c.691C>T | family | asymptomatic |  | metoprolol 25mg/d |
| 7 | female | c.691C>T | family | presyncope | nadolol (1.5mg/kgBW) | - |
| 8 | female | c.1685+2T>G | family | asymptomatic | nadolol (1mg/kgBW) | - |
| 9 | male | c.(449_527)_(860_959)del | family | asymptomatic | propranolol (1mg/kgBW) | - |
| 10 | female | c.1189C>T | unknown | asymptomatic | propranolol  (80mg/d) | metoprolol  (100mg/d) |
| 11 | male | c.477+5G>A | unknown | asymptomatic | propranolol  (80mg/d) | bisoprolol  (1.25mg/d) |
| 12 | male | c.1664G>A | unknown | presyncope | - | metoprolol  (50mg/d) |
| 13 | female | c.1760C>T | proband | asymptomatic | propranolol (0.55mg/kgBW) | - |
| 14 | female | c.724G>A | proband | asymptomatic | propranolol (0.4mg/kgBW) | bisoprolol (2.5mg/d) |
| 15 | male | c.1760C>T | family | asymptomatic | nadolol (0.5mg/kgBW) | atenolol 25mg/d |
| 16 | male | c.1772G>A p | proband | asymptomatic | propranolol (0.8mg/kgBW) |  |

**Supplementary Figure 1**

**12-lead ECG at rest (supine position).**

prior to beta-blocker therapy (noBB, grey; n = 15), during treatment with a non-cardioselective beta-blocker (nsBB, red; n = 14), and during treatment with a cardioselective beta-blocker (sBB, blue; n = 10). Complete ECG data across all three conditions were available for n = 7 participants.

**(A)** RR interval; (**B)** QT interval

*p<0.05, **p<0.01 (mixed-effect analysis for repeated measures with Dunnett`s multiple comparison test)

**C** Scatter plots showing no significant correlation between heart rate and heart rate–corrected Tpeak/end in lead V5 (Tp/e V5 c). Pearson r is -0.16 (p = 0.6).

**Supplementary figure 2**

**Delta Tpeak/end heart-rate corrected at rest and two minutes post exercise**

Without beta-blocker treatment (noBB, grey; n = 14), during treatment with a non-selective beta-blocker (nsBB, red; n = 14), and during treatment with a selective beta-blocker (sBB, blue; n = 10). Complete ECG data across all three conditions were available for n = 6 participants.

Data are shown as individual values with mean ± SD.

**(A)** delta Tpeak/end heart-rate corrected (Tpeak/end c V5 – Tpeak/end c V2) prior to exercise did not differ between the beta-blocker cohorts

**(B)** delta Tpeak/end heart-rate corrected (Tpeak/end c V5 – Tpeak/end c V2) at minute two of the recovery period did not differ between the beta-blocker cohorts

**Supplementary Figure 3**

**Correlation between heart rate and heart rate–corrected Tpeak/end in the nsBB cohort**
**Scatter plots showing no significant correlation between heart rate and heart rate–corrected Tpeak/end in leads V2 (Tp/e V2 c) and V5 (Tp/e V5 c) at rest (A, B) and two minutes after exercise (C, D) in the nsBB cohort.**
(A) Pearson correlation coefficient r = 0.39 (p = 0.33)
(B) Pearson correlation coefficient r = 0.26 (p = 0.41)
(C) Pearson correlation coefficient r = 0.12 (p = 0.68)
(D) Pearson correlation coefficient r = 0.26 (p = 0.46)
